# Effects of therapeutic alliance on patients with major depressive disorder: a literature review

**DOI:** 10.3389/fpsyg.2024.1465017

**Published:** 2025-01-03

**Authors:** Giovanni Videtta, Silvia Busilacchi, Giulia Bartoccioni, Luisa Cirella, Ylenia Barone, Giuseppe Delvecchio

**Affiliations:** ^1^Department of Biomedical Sciences for Health, University of Milan, Milan, Italy; ^2^Department of Neurosciences and Mental Health, Fondazione IRCCS Ca’ Granda Ospedale Maggiore Policlinico, Milan, Italy; ^3^Healthcare Professionals Department, Foundation IRCCS Ca’ Granda Ospedale Maggiore Policlinico, Milan, Italy

**Keywords:** major depressive disorder, therapeutic alliance, clinical setting, clinical psychology, interaction

## Abstract

Therapeutic alliance refers to the collaborative relationship between a therapist and a patient, a concept widely explored in clinical research. It has emerged as a crucial component of effective psychotherapeutic interventions, particularly in the treatment of major depressive disorder (MDD), where its role appears to be multifaceted. In this context, we reviewed the main literature on the role of therapeutic alliance in MDD. The record search was conducted across three databases: PubMed, Web of Science, and PsychInfo. Seven of the reviewed studies highlighted that therapeutic alliance is a strong predictor of clinical outcomes, contributing to symptom improvement, relapse prevention, and more adaptive functioning in patients with MDD. However, three studies supported the hypothesis that clinical improvement itself might influence the therapeutic alliance between a therapist and a patient at different stages of treatment. Overall, the results suggest a bidirectional relationship between therapeutic alliance and symptom improvement, indicating that a stronger alliance often predicts better outcomes and symptom reduction can further enhance the alliance. However, the interpretation of these results must consider certain methodological limitations. These include the use of different approaches, measurements, and clinical outcomes to assess therapeutic alliance, as well as insufficient exploration of the temporal precedence between therapeutic alliance and clinical outcomes. In conclusion, future studies are warranted to address these limitations and further clarify the role of therapeutic alliance in MDD, along with its potential implications for clinical practice.

## 1 Introduction

Therapeutic alliance (TA), also known as the working or helping alliance, is a key concept in psychotherapy, originally defined by Edward Bordin. Therapeutic alliance refers to the collaborative and affective quality of the relationship between a therapist and a patient (Bordin, 1979). An effective TA comprises three essential components: goals, tasks, and the bond between the therapist and the patient. Goals refer to the mutual agreement between the therapist and the patient on the objectives of therapy. Shared goals are crucial as they align both parties on the desired outcome of the therapeutic process, ensuring clarity on the necessary adjustments or improvements needed. Tasks represent the specific activities or interventions agreed upon by both parties to achieve these goals. A clear consensus on tasks enhances the therapeutic process by providing a structured approach to addressing the patient’s needs. Finally, the bond involves the development of a personal connection, trust, and a sense of safety between the therapist and the patient. A strong bond facilitates engagement in therapy, enabling the patient to explore vulnerable topics more comfortably (Bordin, 1979).

Although TA has various definitions in the literature, ranging from the earliest conceptualizations (Freud, 1912; Greenson, 1965; Henry and Strupp, 1994; Hougaard, 1994; Horvath and Luborsky, 1993; Zetzel, 1956) to more recent frameworks (Bender, 2005; Muran and Barber, 2011; Safran and Muran, 2006), its significance has been extensively studied across various psychotherapeutic modalities. A consistent finding is that a strong TA correlates with better clinical outcomes, regardless of the theoretical approach employed (Horvath and Luborsky, 1993).

To better understand the relationship between TA and clinical outcomes, several scales and measurements have been developed to practically and directly assess TA (Ardito and Rebellino, 2011). Among the most widely used, tools that define TA as a “confident collaborative relationship” are the Working Alliance Inventory (WAI), the California Psychotherapy Alliance Scale (CALPAS), the Helping Alliance Questionnaire (HAQ), and the Vanderbilt Therapeutic Alliance Scale (VTAS).

Specifically, the WAI, also available in a short-revised version (WAI-SR), is a patient self-report scale that evaluates the strength and key components of TA (Horvath and Greenberg, 1989; Munder et al., 2010). The CALPAS contains four subscales that assess the TA, the working alliance, the therapist’s contribution to the alliance, and agreement on goals and tasks (Marmar et al., 1986). In contrast, the HAQ, part of the Penn Helping Alliance Scales (PHAS), is a patient self-report tool designed to measure perceptions of the necessary help and collaborative process (Alexander and Luborsky, 1987). Finally, the VTAS evaluates TA by focusing on the relationship between therapist and patient during the psychotherapy process (Suh et al., 1986; Shelef and Diamond, 2008).

Interestingly, additional tools for evaluating TA have been developed in the literature. For example, shorter versions of existing scales have been created to streamline assessments, reduce the burden on patients, and facilitate repeated measurements over time (Paap and Dijkstra, 2017). Additionally, some scales include distinct versions for patients and therapists, limiting the perspective to one member of the therapeutic relationship (Horvath and Greenberg, 1989; Hatcher et al., 2019). This diversity of tools has not only fueled a growing body of research on TA but also addressed ambiguities regarding which factors and aspects are most effective for measuring TA (Paap et al., 2022).

Regardless of its definition, TA has been investigated in several psychopathological disorders, including Major Depressive Disorder (MDD). Indeed, in MDD, building and maintaining an effective TA can be particularly challenging due to the nature of the disorder, which is characterized by apathy, low self-confidence, and difficulties in forming relationships (American Psychiatric Association, 2013). Furthermore, patients with MDD often experience additional symptoms, such as hopelessness, low self-esteem, and challenges in establishing trust, all of which can hinder the development of a strong alliance. To address these barriers, therapists must employ strategies that emphasize empathy, patience, and consistent support (Zuroff et al., 2000; De Bolle et al., 2010).

According to the literature (De Bolle et al., 2010; Zuroff et al., 2000), a strong TA can significantly enhance treatment adherence, improve patient engagement, and increase the overall effectiveness of psychotherapeutic interventions. Moreover, when patients perceive their therapists as supportive and trustworthy, they are more likely to experience meaningful symptom improvements (Zuroff et al., 2000; De Bolle et al., 2010). However, the role of TA in MDD has not been fully explored despite its recognized significance and potential implications for clinical practice. Additionally, TA is not always described as following a linear pattern during psychotherapy; it can be easily disrupted throughout therapy. Notably, studies have shown that positive therapeutic outcomes are often correlated with the successful resolutions of ruptures in the alliance (Safran et al., 1990; Safran and Muran, 1996).

Therefore, this review aims to explore and evaluate the pivotal role of TA in MDD, encapsulating main evidence on those factors that might contribute to the relationship between TA and clinical outcome.

## 2 Materials and methods

**Figure 1:**
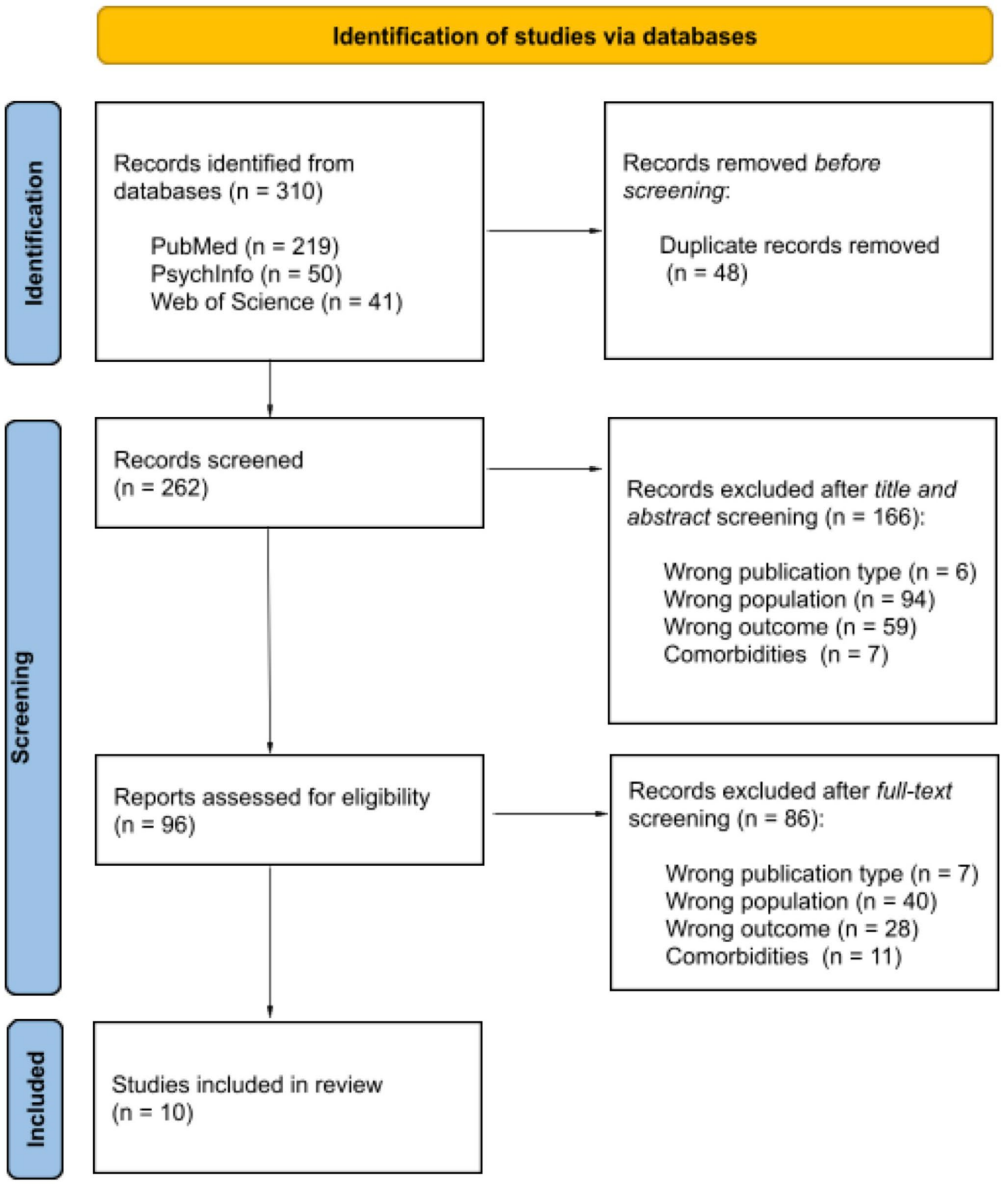
PRISMA flowchart diagram for record selection (adapted from: Page et al., 2021, doi: 10.1136/bmj.n71).

Records were searched on three datasets: PubMed, Web of Science, and PsychInfo. The following research string was used: therapeutic alliance OR helping alliance OR working alliance AND (clinician OR therapist OR psychotherapist OR psychologist) AND (instrument OR measure OR questionnaire) AND major depressive disorder. To be included, a paper (i) was a peer-reviewed original publication, (ii) was written in the English language, (iii) involved patients with MDD diagnosis, and (iv) employed a standardized tool for evaluating TA. A study was excluded if (i) patients showed neurological and/or psychiatric comorbidities, (ii) the mean age of patients was under 18 years or over 50 years, and (iii) the experimental design was cross-sectional. The clinical treatment, the theoretical background of therapists, the duration of the therapy, and the recruitment setting (e.g., inpatients and outpatients) were not limited. The search of records was performed on the 28th of March 2024, and no temporal window was set. Figure 1 reported the flowchart describing the record selection. Out of 310 records, 262 were unique records. At the end of the screening by title and abstract, 96 were full-text read, and only 10 matched our selection criteria. In Table 1, the included records, the extracted variables, and the main results are reported.

**Table 1:** Sociodemographic and clinical characteristics of the reviewed studies.

Study	Sample size (M/F)	Age (mean ± sd)	Clinical variables					Main results
			Clinical treatment	Theoretical background	Duration of psychotherapy	Tool for TA (evaluation time points)	Recruitment setting	
Berger et al. (2018)	98 (33/65)	43.1 ± 12.0	CBT Psychodynamic psychotherapy Humanistic psychotherapy Integrative psychotherapy Other	20 psychotherapists	12 wk	WAI-SR (6 wk, 12 wk)	Outpatients	TA predicts clinical outcomes (partially).
Castonguay et al. (1996)	30 (7/23)	33.8 ± −	CBT Pharmacotherapy	psychologist social workers	12 wk	WAI (weekly)	Outpatients	TA predicts clinical outcomes.
De Bolle et al. (2010)	567 (188/379)	39.5 ± 10.6	Supportive psychotherapy CBT Psychodynamic psychotherapy Pharmacotherapy	141 psychiatrists	24 wk	HAQ (4 wk)	Outpatients	TA predicts clinical outcomes.
Feeley et al. (1999)	25 (3/22)	32.9 ± 11.2	CBT Pharmacotherapy	4 psychotherapists 3 social workers 1 psychologist	12 wk	PHAS (−)	Inpatients	Symptom change predicts TA.
Gibbons et al. (2019)	237 (59/178)	36.2 ± 12.1	SEDT CBT	20 clinicians	20 wk	WAI (≅1 wk)	Outpatients	TA predicts treatment outcome (duration).
Stiles-Shields et al. (2014)	325 (170/155)	47.7 ± 13.1	CBT	291 psychologists	18 wk	WAI (4 wk, 14 wk)	Outpatients	TA predicts clinical outcomes.
Strunk et al. (2010)	60 (25/35)	40.0 ± 12.0	CBT	5 psychologists 1 psychiatric nurse	–	WAI (1 wk, 2 wk, 3 wk, 4 wk)	Outpatients	Symptom change predicts TA.
Weck et al. (2013)	80 (25/55)	48.3 ± 11.6	CBT	25 psychologists 1 physician	32 wk	HAQ (weekly)	Outpatients	TA predicts relapse time.
Wucherpfennig et al. (2017)	211 (70/141)	38.0 ± 12.7	CBT	89 psychotherapists	- (≅ 37 sessions)	BPSR (after each session)	Outpatients	Gains and treatment outcomes predict TA.
Zuroff and Blatt (2006)	191 (61/130)	35.2 ± 8.5	CBT IPT Pharmacotherapy	–	16 wk	B-L RI (4 wk) VTAS (8 wk)	Outpatients	TA predicts clinical adaptation.

–, not measured and/or not reported; B-L RI, Barrett-Lennard Relationship Inventory; BPSR, Bern Postsession Reports; CBT, Cognitive Behavioral Therapy; F, Females; HAQ, Helping Alliance Questionnaire; IPT, Interpersonal Therapy; M, Males; MDD, Major Depressive Disorder; PHAS, Penn Helping Alliance Scale; sd, Standard Deviation; SEDT, Supportive Dynamic psychoTherapy; TA, Therapeutic Alliance; VTAS, Vanderbilt Therapeutic Alliance Scale; WAI, Working Alliance Inventory; WAI-SR, Working Alliance Inventory Short Revised; wk, Weeks.

## 3 Results

Out of ten studies, six of them (Berger et al., 2018; Castonguay et al., 1996; De Bolle et al., 2010; Feeley et al., 1999; Gibbons et al., 2019; Zuroff and Blatt, 2006) used multiple clinical treatments, while four (Stiles-Shields et al., 2014; Strunk et al., 2010; Weck et al., 2013; Wucherpfennig et al., 2017) focused on a single treatment. Regarding standardized tools for measuring TA, five studies used the WAI (Gibbons et al., 2019; Stiles-Shields et al., 2014; Strunk et al., 2010; Berger et al., 2018; Castonguay et al., 1996); two studies used the HAQ (De Bolle et al., 2010; Weck et al., 2013); one study used the PHAS (Feeley et al., 1999); one study used the Bern Postsession Reports (BPSR) (Wucherpfennig et al., 2017); and, finally, one study used both the Barrett-Lennard Relationship Inventory (B-L RI) and VTAS (Zuroff and Blatt, 2006). Notably, all reviewed studies included MDD outpatients, except for Feeley et al. (1999), which focused on MDD inpatients.

Finally, seven studies (Berger et al., 2018; Castonguay et al., 1996; De Bolle et al., 2010; Gibbons et al., 2019; Stiles-Shields et al., 2014; Weck et al., 2013; Zuroff and Blatt, 2006) identified TA as a predictor of clinical outcomes in MDD, while three (Feeley et al., 1999; Strunk et al., 2010; Wucherpfennig et al., 2017) reported clinical improvement as a predictor of TA.

### 3.1 Therapeutic alliance as a predictor of clinical outcomes

Seven studies showed that TA predicted clinical outcomes in MDD patients (Berger et al., 2018; Castonguay et al., 1996; De Bolle et al., 2010; Gibbons et al., 2019; Stiles-Shields et al., 2014; Weck et al., 2013; Zuroff and Blatt, 2006). Four studies included only psychotherapeutic treatment (Berger et al., 2018; Gibbons et al., 2019; Stiles-Shields et al., 2014; Weck et al., 2013), while three studies (Castonguay et al., 1996; De Bolle et al., 2010; Zuroff and Blatt, 2006) offered a combined treatment of psychotherapy and pharmacotherapy. However, even among the studies that did not include pharmacological treatment, some participants also received medication. In the study by Stiles-Shields et al. (2014), approximately 30% of the patients took an antidepressant. This percentage reached approximately 50% in the study by Berger et al. (2018). In the research conducted by Weck et al. (2013), approximately 65% of the participants received antidepressant treatment. On the other hand, Gibbons et al. (2019) did not provide information on whether the participants took any pharmacological treatment.

Specifically, Berger et al. (2018) recruited 98 MDD outpatients who were divided into two psychotherapy groups: face-to-face (FtF) and combined (FtF and a web-based treatment tool). In each group, 20 psychotherapists applied different clinical treatments, including cognitive-behavioral therapy (CBT), psychodynamic psychotherapy, humanistic psychotherapy, and integrative psychotherapy, for a period of 12 weeks. WAI-SR Client Version (WAI-SR-C) and WAI-SR Therapist Version (WAI-SR-T) were administered after 6 weeks and 12 weeks from the beginning of the treatment. The authors found that at 6 weeks and 12 weeks, WAI-SR-C scores predicted the clinical outcome in FtF psychotherapy and combined psychotherapy groups (only at 12 weeks).

Another piece of interesting evidence comes from Stiles-Shields et al. (2014), who investigated TA in two different CBT treatments: FtF-CBT and telephone CBT (T-CBT). The MDD sample comprised 325 outpatients, while the therapists were 291 psychologists. The duration of the treatment was 18 weeks, and WAI was employed to measure TA after 4 and 14 weeks of treatment. The authors found that in both FtF-CBT and T-CBT treatment, higher WAI scores were correlated to a reduction of depressive symptoms during the entire duration of treatment.

Investigating TA and the risk of MDD relapse, Weck et al. (2013) designed an experiment involving 80 MDD outpatients, 25 psychologists, and 1 physician. Patients underwent CBT treatment for 16 therapy sessions within 8 months, and after each therapy session, TA was measured with HAQ. An interesting result was that increasing values for TA, measured as the HAQ mean score aggregate over all 16 therapy sessions, were associated with a decrease in the risk of new depressive episodes, generally 1 year after the treatment. In addition, high TA values reduced the risk of relapse for patients with five or more previous depressive episodes. On the other hand, low TA values correlated with an increase in the risk of a relapse for the same patients.

In another study, Gibbons et al. (2019) highlighted how the quality of TA impacted both the relationship between clinician and patient and the duration of clinical treatment. A sample of 237 MDD outpatients received a 20-week treatment by 20 clinicians who applied supportive-expressive dynamic therapy (SEDT) and CBT. The authors measured TA with WAI, which was administered after the second therapy session, approximately 1 week after starting treatment. The main result was that patients with low agreement on the tasks were more likely to terminate the treatment very early, after only two to six therapy sessions, and this result was reported in both CBT and SEDT.

Similar results were found by Castonguay et al. (1996), who investigated TA in 30 MDD outpatients, who were followed by one psychologist and three social workers and received treatment for 12 weeks. Two clinical treatments were applied; one group received only CBT, while the other group was offered a combined treatment: CBT and pharmacotherapy. TA was measured with WAI at the end of each weekly therapy session. Despite the two experimental conditions, the two groups were merged in the analyses, controlling for the type of treatment, and TA was assessed by the professional providing the psychotherapeutic treatment. The authors reported that TA was significantly related to clients’ improvement in depressive symptoms and global functioning at 6 and 12 weeks.

De Bolle et al. (2010) investigated TA between 144 psychiatrists and 567 MDD outpatients who underwent a 24-week treatment. The study included various experimental conditions based on the combination of three psychotherapeutic approaches (supportive psychotherapy, CBT, and psychodynamic psychotherapy) and two pharmacological treatments. The psychiatrists applied the clinical treatments, who also administered the medication in a double-blind design. During the fourth week of treatment, TA was measured using HAQ. The authors reported that HAQ scores positively predicted the clinical change, controlling for the effect of early improvement.

Finally, Zuroff and Blatt (2006) recruited 191 MDD outpatients who were assigned to one of four experimental conditions: two involved psychotherapeutic treatment (CBT, interpersonal therapy), and two involved pharmacological treatment with clinical management. The duration of treatment was 16 weeks, and TA was measured by using B-L RI, administered after 4 weeks of treatment, and VTAS, administered after 8 weeks of treatment. The authors reported that in all clinical treatments, the perceived quality of an early therapeutic relationship, including TA, adjusted for early clinical improvement, predicted the rate of decrease in maladjustment after measuring the relationship. Moreover, high-quality early relationships predicted lower levels of maladjustment throughout the 18-month follow-up and higher levels of adaptive capacities.

### 3.2 Clinical improvement as a predictor of a therapeutic alliance

Three studies (Feeley et al., 1999; Strunk et al., 2010; Wucherpfennig et al., 2017) reported that clinical improvement predicted the TA. Of these, only one study (Feeley et al., 1999) included two experimental conditions: CBT alone or CBT combined with pharmacological treatment. However, since no differences were found in the pattern of results, the authors reported that all analyses with the two groups had merged. In the study by Strunk et al. (2010), patients were offered only cognitive therapy (CT), while in the study by Wucherpfennig et al. (2017), no data were provided regarding the participants’ pharmacological treatment.

Feeley et al. (1999) recruited 25 MDD inpatients and involved four psychotherapists, three social workers, and one psychologist. Patients followed a 12-week treatment, and two clinical treatments were applied: CBT and pharmacotherapy. TA was measured by PHAS. The authors reported that the TA at the first sessions did not predict any symptomatology changes in the following sessions. Moreover, the researchers presented evidence regarding symptom changes during treatment and possible effects on the TA. They found that changes in MDD symptoms did not appear to predict higher PHAS scores, as the results only revealed a trend, rather than statistical significance.

Similar findings were reported by Strunk et al. (2010), who recruited 60 MDD outpatients treated by five psychologists and one psychiatric nurse. The patients received CBT treatment, and TA was measured weekly over 4 weeks using the WAI. The authors found that TA was not a significant predictor of symptom scores during therapy sessions. Instead, prior symptom improvement predicted TA scores, with higher TA scores following greater symptom improvement.

Similarly, Wucherpfennig et al. (2017) examined 211 MDD outpatients who underwent approximately 37 CBT sessions with 89 psychotherapists. TA was measured at the end of each session using the BPSR. The study found that TA increased following a “sudden gain,” defined as a meaningful clinical improvement between therapy sessions. In addition, patients who experienced sudden gains tended to achieve higher TA levels more quickly than those who did not.

## 4 Discussion

The reviewed studies showed that TA can be a good predictor of clinical outcomes, while clinical improvement during psychotherapy can also impact TA.

Interestingly, the relationship between TA and clinical outcomes did not appear to be affected by factors such as the clinical setting, treatment type, or the therapist’s theoretical orientation. These results are in line with existing literature that highlights TA as a critical determinant of treatment outcomes in MDD, regardless of sample size, method of alliance assessment, therapy duration, or specific therapeutic techniques (Flückiger et al., 2018; Martin et al., 2000; Horvath and Symonds, 1991). Moreover, an initial high level of common factor techniques, such as empathy, active listening, hope, and encouragement, has been shown to predict a stronger alliance later in therapy. In turn, a stronger alliance makes the continued use of these common factor techniques more likely (Solomonov et al., 2018).

As mentioned above, seven reviewed studies (Berger et al., 2018; Castonguay et al., 1996; De Bolle et al., 2010; Stiles-Shields et al., 2014; Zuroff and Blatt, 2006; Gibbons et al., 2019; Weck et al., 2013) found that TA predicted clinical outcomes in MDD patients. These findings emphasize that a strong therapeutic relationship between therapist and patient is crucial for countering premature treatment termination, facilitating symptom improvement, and promoting more adaptive functioning. This evidence supports the idea that the therapeutic relationship is not only necessary for implementing specific techniques (Beck et al., 1979) but is inherently therapeutic in itself (Rogers, 1957; Zilcha-Mano, 2017).

Furthermore, the quality of TA in MDD, even in the absence of face-to-face interaction (Berger et al., 2018; Stiles-Shields et al., 2014), aligns with research demonstrating no significant differences in alliance outcomes between traditional psychotherapy and internet-based or telephone-based psychotherapy (Flückiger et al., 2018; Sucala et al., 2012; Jenkins-Guarnieri et al., 2015; Reynolds and Stiles, 1982). Notably, several studies have also highlighted that in MDD, a patient’s perception of TA tends to be a stronger predictor of clinical outcomes compared to assessments made by therapists or external observers (Berger et al., 2018; Zuroff and Blatt, 2006), as similarly noted in the broader literature (Martin et al., 2000; Horvath and Symonds, 1991; Flückiger et al., 2018).

Furthermore, in MDD, the relationship between TA and clinical outcomes was highlighted in studies that correlated alliance measures with the total reduction of symptoms during treatment (Stiles-Shields et al., 2014; Berger et al., 2018; De Bolle et al., 2010; Castonguay et al., 1996; Zuroff and Blatt, 2006). These findings align with the broader literature, which consistently shows that higher alliance scores predict better treatment outcomes (Horvath and Symonds, 1991; Horvath et al., 2011; Martin et al., 2000; Flückiger et al., 2018).

An intriguing observation from the reviewed studies is that the relationship between TA and clinical outcomes was evident both when standardized TA tools were administered at specific time points (Stiles-Shields et al., 2014; Berger et al., 2018; Gibbons et al., 2019) and when the average score of TA measurements across sessions was used (Weck et al., 2013). This finding is partially supported by existing literature, which suggests that an average alliance score may be a more reliable predictor of outcomes than single-session TA assessments (Crits-Christoph et al., 2011).

Finally, three reviewed studies (Feeley et al., 1999; Strunk et al., 2010; Wucherpfennig et al., 2017) highlighted that clinical outcome predicted TA. Specifically, Strunk et al. (2010) and Wucherpfennig et al. (2017) found higher alliance scores following greater symptom improvement, while Feeley et al. (1999) revealed a positive relationship between previous symptom improvement and TA. However, this finding did not reach statistical significance. These results support the concept of a reverse relationship, suggesting that a strong TA may develop as a result of symptomatic changes (Puschner et al., 2008).

One possible explanation is that symptom reduction may enhance the patient’s trust in the treatment and the therapist, thereby strengthening the alliance. From the therapist’s perspective, it is also plausible that they perceive the alliance as stronger with patients who appear to benefit from the treatment. However, the hypothesis that symptomatic improvement leads to a stronger therapeutic alliance is not entirely supported by the literature. Mixed findings have been reported regarding the relationship between improvements in alliance and subsequent symptom changes (Crits-Christoph et al., 2011; Hendriksen et al., 2014; Barber et al., 2000).

Importantly, interpreting the results of this review requires consideration of several methodological limitations. First, the reviewed studies used diverse approaches to investigate the alliance-outcome relationship, complicating direct comparisons of their conclusions. For instance, while some studies employed traditional clinical treatments to evaluate and strengthen TA, others relied on unconventional methods, such as telephone-based interventions. Additionally, in several studies, patients received pharmacological treatment, which served as a potential confounding variable that was not always accounted for by the researchers.

Second, TA was measured at varying time points or during every therapy session, introducing inconsistencies in the assessment process. Third, outcome measures differed among the reviewed studies, further complicating comparisons. Finally, temporal precedence, distinguishing between clinical changes occurring before or after the evaluation of TA, was poorly investigated.

In conclusion, the findings suggest that in MDD patients, TA is a reliable predictor of clinical outcomes, contributing to symptom improvement, relapse prevention, and more adaptive functioning. However, this literature review does not fully clarify whether clinical improvement directly influences TA. It is possible that the relationship between therapist and patient is shaped by prior intersubjective conditions, which may enhance clinical benefits while supporting the development of a strong and effective TA. Future research is needed to address the limitations highlighted in this review by reducing methodological heterogeneity in approaches, measurements, and outcome variables. Additionally, a deeper investigation into temporal precedence is warranted. These efforts could provide greater insights into the relationship between TA and clinical outcomes in MDD, ultimately supporting the development of more effective treatments for depression.
